# Monocyte-to-lymphocyte ratio as a determinant of survival in patients with gastric cancer undergoing gastrectomy: A cohort study

**DOI:** 10.1097/MD.0000000000033930

**Published:** 2023-06-02

**Authors:** Soomin An, Wankyu Eo, Sookyung Lee, Yeong-Ju Lee

**Affiliations:** a Department of Nursing, Dongyang University, Gyeongbuk, Republic of Korea; b College of Medicine, Kyung Hee University, Seoul, Republic of Korea; c Department of Clinical Oncology, College of Korean Medicine, Kyung Hee University, Seoul, Republic of Korea; d College of Nursing Science, Kyung Hee University, Seoul, Republic of Korea.

**Keywords:** gastrectomy, inflammation, lymphocyte, monocyte, prognosis, stomach neoplasms

## Abstract

The monocyte-to-lymphocyte ratio (MLR) is an important prognostic determinant of various malignancies. However, the prognostic role of MLR in patients with gastric cancer undergoing gastrectomy remains unclear. Patients with stage I to III gastric cancer who underwent curative-intent gastric resection were enrolled in this study. Cox regression analysis was used to determine the independent variables for overall survival (OS) and disease-free survival (DFS). The established models were validated internally. Inter-model comparisons were performed using the integrated area under the receiver operating characteristic curve and the concordance index. Multivariate Cox regression analysis revealed that age, tumor–node–metastasis (TNM) stage, perineural invasion, serum albumin level, and MLR were prognostic factors for OS and DFS and constituted the full model. The full model was internally validated using calibration curves and decision curve analysis. The integrated area under the curve and concordance index of the full model outperformed those of TNM stage. The full model was a significant determinant of OS and DFS. Additionally, the full model was suggested to outperform TNM stage in predicting patient survival outcomes.

## 1. Introduction

Gastric cancer (GC) is a major health problem owing to its high incidence, frequent late presentation, and poor survival rates. This disease is more common in older adults, predominantly men. Surgical resection is the primary treatment for patients with stage I to III GC. However, the recurrence rate remains high, leading to death.^[1]^ Therefore, several predictive models have been developed to identify patients with stage I to III GC who are at the highest risk of future relapse or death.^[2]^

The tumor–node–metastasis (TNM) staging system is considered the gold standard for predicting the risk of future relapse or death. However, the TNM staging system has some limitations such as the inability to incorporate tumors, nodes, or metastases as continuous variables. In addition, they do not account for molecular characteristics, such as gene mutations or specific protein expression, which can have significant implications for prognosis and treatment.^[3]^ Moreover, it does not consider other factors that can affect a patient’s overall health such as age, overall health status, or other comorbid conditions.^[3]^ Given the limitations of the TNM system, it is necessary to establish a novel predictive model that is simple and advantageous.

Serum albumin level (ALB), an indicator of nutritional status, is known to play a significant role in the prognosis of GC. Low levels of ALB have been linked to an increased risk of postoperative infectious complications and poorer survival outcomes in GC patients.^[4]^ Recently, the geriatric nutritional risk index has emerged as a potential determinant of survival outcomes; however, further external validation is needed.^[5]^

Tumors can stimulate the production and release of monocytes from the bone marrow into the bloodstream, leading to an increase in absolute monocyte count (AMC). This increase in AMC can contribute to the development and progression of malignancy by promoting a supportive microenvironment for tumor growth, angiogenesis, and recruitment of other immune cells.^[6–8]^ Additionally, tumors can directly or indirectly affect the immune system, leading to a decrease in lymphocyte production or function. The tumor microenvironment can also play a role in inducing lymphocytopenia, which can further attenuate the host response to tumors, promoting tumor progression and metastasis.^[8]^ Therefore, models that incorporate both AMC and absolute lymphocyte count (ALC) can help understand the interaction between the immune system and cancer. However, the optimal cutoff points for several proposed models, such as the absolute monocyte and lymphocyte count prognostic score^[9]^ and lymphocyte-to-monocyte ratio,^[10]^ have not been agreed upon, limiting their clinical utility in patients with GC.

Recently, the monocyte-to-lymphocyte ratio (MLR) has garnered attention as a biomarker that is elevated in various disease conditions, including diabetic nephropathy,^[11]^ gastrointestinal disorders,^[12]^ frailty,^[13]^ and COVID-19 infection.^[14]^ Moreover, the MLR has been found to be a significant prognostic factor in various malignancies, including hepatocellular carcinoma,^[15]^ gallbladder carcinoma,^[16]^ esophagogastric junction cancer,^[17]^ gastrointestinal stromal tumor,^[18]^ colorectal cancer^[19–21]^ and GC.^[22–31]^

However, the optimal cutoff values for MLR in patients with GC undergoing curative-intent resection remain unclear, and there is limited multivariate analysis to support its clinical value.^[27–31]^ To address this gap, we evaluated the clinical value of MLR in our patient cohort by treating variables as continuous without dichotomization to prevent overfitting. We also employed both univariate and multivariate Cox regression analyses to assess the clinical significance of variables.

## 2. Methods

### 2.1. Patients

Electronic medical records of consecutive patients with GC who underwent curative-intent surgical resection at Kyung Hee University Hospital at Gangdong from June 2006 to October 2017 were reviewed. The inclusion criteria were as follows: Stage I to III GC;^[32]^ No preoperative anticancer treatment; and Microscopically margin-negative resection (i.e., resection). The exclusion criteria were as follows: Concurrent malignancies, or prior malignancies within the last 5 years; Microscopic or macroscopic residual tumor; and Autoimmune diseases or active infection.

This study was approved by the Institutional Review Board of Kyung Hee University Hospital (IRB File No. 2021-06-021), which waived the requirement for individual consent.

### 2.2. Baseline clinical characteristics

Demographic parameters included age, sex, and body mass index. Pathological parameters included tumor site, tumor size, extent of the primary tumor (T stage), presence or absence of cancer cells in nearby lymph nodes (N stage), TNM stage, gastrectomy type, histological classification, perineural invasion (PNI), lymphatic invasion, and vascular invasion.

Blood test results obtained within 7 days prior to surgery were analyzed, and if multiple test results were available, those closest to the surgery date were selected. Hematological parameters including leukocyte and differential counts (AMC, ALC, and absolute neutrophil count), hemoglobin concentration, and platelet count, were processed at room temperature within 1 hour of venipuncture according to local guidelines.^[33,34]^ Inflammatory parameters, such as MLR, neutrophil-to-lymphocyte ratio (NLR), and platelet-to-lymphocyte ratio (PLR), were calculated using absolute neutrophil count, AMC, ALC, and platelet count. The MLR was defined as follows: MLR = (AMC **× **10)/ALC. In this study, ALB was evaluated as the nutritional parameter.

### 2.3. Statistical analysis

We assessed the normality of continuous variables using the Shapiro–Wilk test. However, to mitigate the impact of extreme values or outliers and to prevent potential bias, we reported the median values with interquartile range instead of the mean and standard deviation for all continuous variables.

Overall survival (OS) was defined as the time interval between the date of surgery and death from any cause, whereas disease-free survival (DFS) was defined as the time interval between the date of surgery and first the documented evidence of disease recurrence or death from any cause, whichever occurred first.

A prediction model was developed using the entire dataset. Cox regression analysis was performed only for the variables that met Schoenfeld assumption of proportional hazards. Variance inflation factors (VIF) of the covariates were calculated to determine multicollinearity.

Nomograms were constructed using established models. The performance and optimism of the developed models were evaluated by using a bootstrapping technique with resampling. Bootstrapping provides a nearly unbiased estimate of the prediction accuracy using a large number of samples from the original sample.^[35]^ Calibration was performed with 1000 bootstrap samples for internal validation of the models while avoiding overfitting. In addition, decision curve analysis (DCA) with 500 bootstrap samples was performed for the internal validation of the models.

The time-dependent area under the curve (AUC), which is also referred to as AUC (t), of the models was determined at 36 and 60 months after surgical resection. Additionally, the model’s AUC (t) was plotted over 10 years after surgical resection, using an incident/dynamic approach.

The integrated AUC (iAUC) and concordance index (C-index) of the models were used to assess the model discrimination.^[36]^ The difference in c-index between the models was calculated as previously reported.^[37]^

All *P* values presented were 2-sided, and statistical significance was set at *P* < .05. Statistical analyses were performed using the R package.

## 3. Results

### 3.1. Clinical characteristics of patients

Of the 405 patients assessed for eligibility, 3 patients with concurrent malignant tumors, 1 patient with microscopic residual disease, and 1 patient with stage IV disease were excluded. Therefore, a total of 400 patients were included in the final analysis. The clinical characteristics of the patients are summarized in Table 1. The median age of the patients was 61 years, and 38 patients (9.5%) had early onset GC. There were more men (66.5%) than women in the present study. In total, 244 (61.0%), 74 (18.5%), and 82 (20.5%) patients had stage I, II, and III GC, respectively. Intestinal type was the most common (49.2%), and PNI was detected in 34 (8.5%) patients. Anemia and hypoalbuminemia were diagnosed in 147 (36.8%) and 30 (7.5%) patients, respectively. With the exception of age, body mass index, tumor size, ALB, NLR, PLR, and MLR were found to have non-normal distributions, as determined by the Shapiro–Wilk test.

**Table 1:** Characteristic of patients.

Variables	Median (IQR); or *n* (%)
Age, yr	61 (52–70)
Sex	
Male	266 (66.5%)
Female	134 (33.5%)
BMI, kg/m^2^	23.6 (21.3–25.8)
Site of tumor	
Upper	41 (10.2%)
Middle	164 (41.0%)
Lower	189 (47.2%)
Diffuse	6 (1.5%)
Size of tumor, cm	3.0 (2.0–5.5)
T stage	
1–2	270 (67.5%)
3–4	130 (32.5%)
N stage	
0	260 (65.0%)
1–2	140 (35.0%)
TNM stage	
I	244 (61.0%)
II	74 (18.5%)
III	82 (20.5%)
Type of gastrectomy	
Partial	316 (79.0%)
Total	84 (21.0%)
Lauren classification	
Intestinal	197 (49.2%)
Diffuse	96 (24.0%)
Mixed	92 (23.0%)
Unknown	15 (3.8%)
Perineural invasion	
No	366 (91.5%)
Yes	34 (8.5%)
Lymphatic invasion	
No	267 (66.8%)
Yes	133 (33.2%)
Vascular invasion	
No	380 (95.0%)
Yes	20 (5.0%)
White blood cell, per μL	6500 (5395–7850)
Anemia*	
No	253 (63.2%)
Yes	147 (36.8%)
Platelet, × 10^3^/μL	235.5 (203.0–278.0)
Serum albumin, g/dL	4.1 (3.9–4.3)
Adjuvant chemotherapy	
No	255 (63.7%)
Yes	145 (36.2%)

BMI = body mass index, IQR = interquartile range, N stage = presence or absence of cancer cells in nearby lymph nodes, T stage = extent of the primary tumor, TNM = tumor–node-metastasis.

* The cutoff point was 12 g/dL in female patients and 13 g/dL in male patients.

### 3.2. Cox regression analysis of the risk factors on survival

The median follow-up time was 72.6 months (interquartile range: 29.9–97.1 months). The probability of OS at 5 years was 80.2%, whereas that of DFS at 5 years was 77.3%.

In univariate Cox regression analysis, age, tumor size, N stage, TNM stage, PNI, lymphatic invasion, vascular invasion, anemia, ALB, NLR, PLR, and MLR were found to be significant variables for OS. In multivariate Cox regression analysis for OS, age (hazard ratio [HR] 1.04, *P < *.001), TNM stage (HR 3.87, *P < *.001), PNI (HR 2.22, *P = *.011), ALB (HR 0.34, *P < *.001), and MLR (HR 1.23, *P = *.008) were significant variables, with VIFs ranging from 1.12 to 1.21 (Table 2).

**Table 2:** Univariate and multivariate Cox regression analyses of overall survival.

Variable	Univariate		Multivariate	
	HR (95% CI)	*P* value	HR (95% CI)	*P* value
Age (yr)*	1.06 (1.03–1.08)	<.001	1.04 (1.02–1.06)	<.001
Sex (female vs male)	0.75 (0.47–1.20)	.234		
BMI (kg/m^2^)*	0.95 (0.88–1.01)	.097		
Size of tumor (cm)*	1.18 (1.13–1.23)	<.001		
N stage (1–2 vs 0)	3.60 (2.33–5.56)	<.001		
TNM stage (III vs I–II)	5.67 (3.70–8.69)	<.001	3.87 (2.45–6.11)	<.001
Histology (intestinal vs others)†	0.91 (0.60–1.39)	.671		
Perineural invasion (yes vs no)	2.74 (1.54–4.89)	<.001	2.22 (1.20–4.10)	.011
Lymphatic invasion (yes vs no)	3.62 (2.35–5.57)	<.001		
Vascular invasion (yes vs no)	3.42 (1.77–6.63)	<.001		
Anemia (yes vs no)‡	3.28 (2.13–5.96)	<.001		
Albumin (g/dL)*	0.20 (0.14–0.29)	<.001	0.34 (0.22–0.52)	<.001
Neutrophil-to-lymphocyte ratio*	1.23 (1.11–1.35)	<.001		
Platelet-to-lymphocyte ratio*	1.01 (1.00–1.01)	<.001		
Monocyte-to-lymphocyte ratio*	1.46 (1.30–1.64)	<.001	1.23 (1.06–1.43)	.008

BMI = body mass index, CI = confidence interval, HR = hazard ratio, N stage = presence or absence of cancer cells in nearby lymph nodes, TNM = tumor–node–metastasis.

* Continuous variable.

† Lauren classification.

‡ Cutoff point is 12 g/dL in female patients and 13 g/dL in male patients.

Similarly, age, TNM stage, PNI, ALB, and MLR were significant variables in DFS analysis, as determined by multivariate Cox regression analysis. Their respective HRs and *P* values were HR 1.01 and *P* = .004 for age, HR 4.44 and *P* < .001 for TNM stage, HR 1.86 and *P* = .039 for PNI, HR 0.31 and *P* < .001 for ALB, and HR 1.23 and *P* = .003 for MLR. The VIFs for these variables ranged from 1.10 to 1.28 (Table 3).

**Table 3:** Univariate and multivariate Cox regression analyses of disease-free survival.

Variable	Univariate		Multivariate	
	HR (95% CI)	*P* value	HR (95% CI)	*P* value
Age (yr)*	1.05 (1.03–1.07)	<.001	1.01 (1.01–1.05)	.004
Sex (female vs male)	0.64 (0.41–1.01)	.057		
BMI (kg/m^2^)*	0.95 (0.90–1.01)	.135		
Size of tumor (cm)*	1.18 (1.14–1.23)	<.001		
N stage (1–2 vs 0)	4.08 (2.69–6.20)	<.001		
TNM stage (III vs I–II)	6.06 (4.03–9.08)	<.001	4.44 (2.88–6.84)	<.001
Histology (intestinal vs others)†	0.93 (0.62–1.38)	.708		
Perineural invasion (yes vs no)	2.59 (1.49–4.51)	<.001	1.86 (1.03–3.35)	.039
Lymphatic invasion (yes vs no)	3.55 (2.36–5.33)	<.001		
Vascular invasion (yes vs no)	4.10 (2.23–7.51)	<.001		
Anemia (yes vs no)‡	3.13 (2.08–4.71)	<.001		
Albumin (g/dL)*	0.17 (0.11–0.24)	<.001	0.31 (0.20–0.50)	<.001
Neutrophil-to-lymphocyte ratio*	1.23 (1.13–1.34)	<.001		
Platelet-to-lymphocyte ratio*	1.01 (1.00–1.01)	<.001		
Monocyte-to-lymphocyte ratio*	1.44 (1.30–1.59)	<.001	1.23 (1.08–1.41)	.003

BMI = body mass index, CI = confidence interval, HR = hazard ratio, N stage = presence or absence of cancer cells in nearby lymph nodes, TNM = tumor–node–metastasis.

* Continuous variable.

† Lauren classification.

‡ Cutoff point is 12 g/dL in female patients and 13 g/dL in male patients.

### 3.3. Establishment and validation of the model for survival

**Figure 1.**
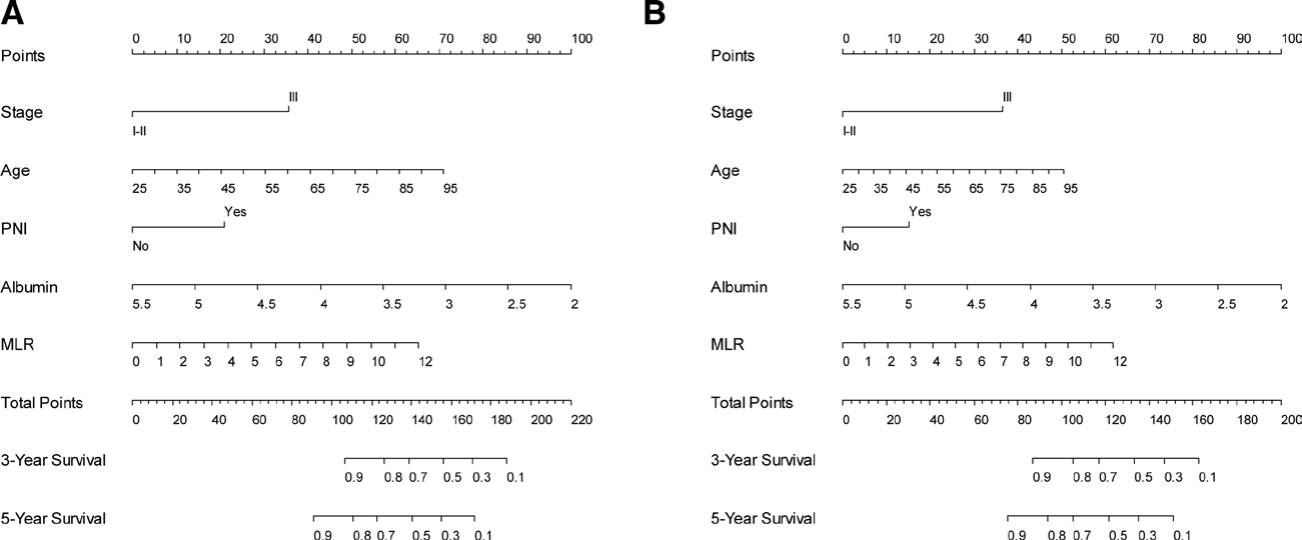
Nomograms of full model. (A) Overall survival, (B) disease-free survival. Full model = the model consisting of age, TNM stage, PNI, serum albumin level, and MLR. MLR = monocyte-to-lymphocyte ratio, PNI = perineural invasion, TNM = tumor–node–metastasis.

**Figure 2.**
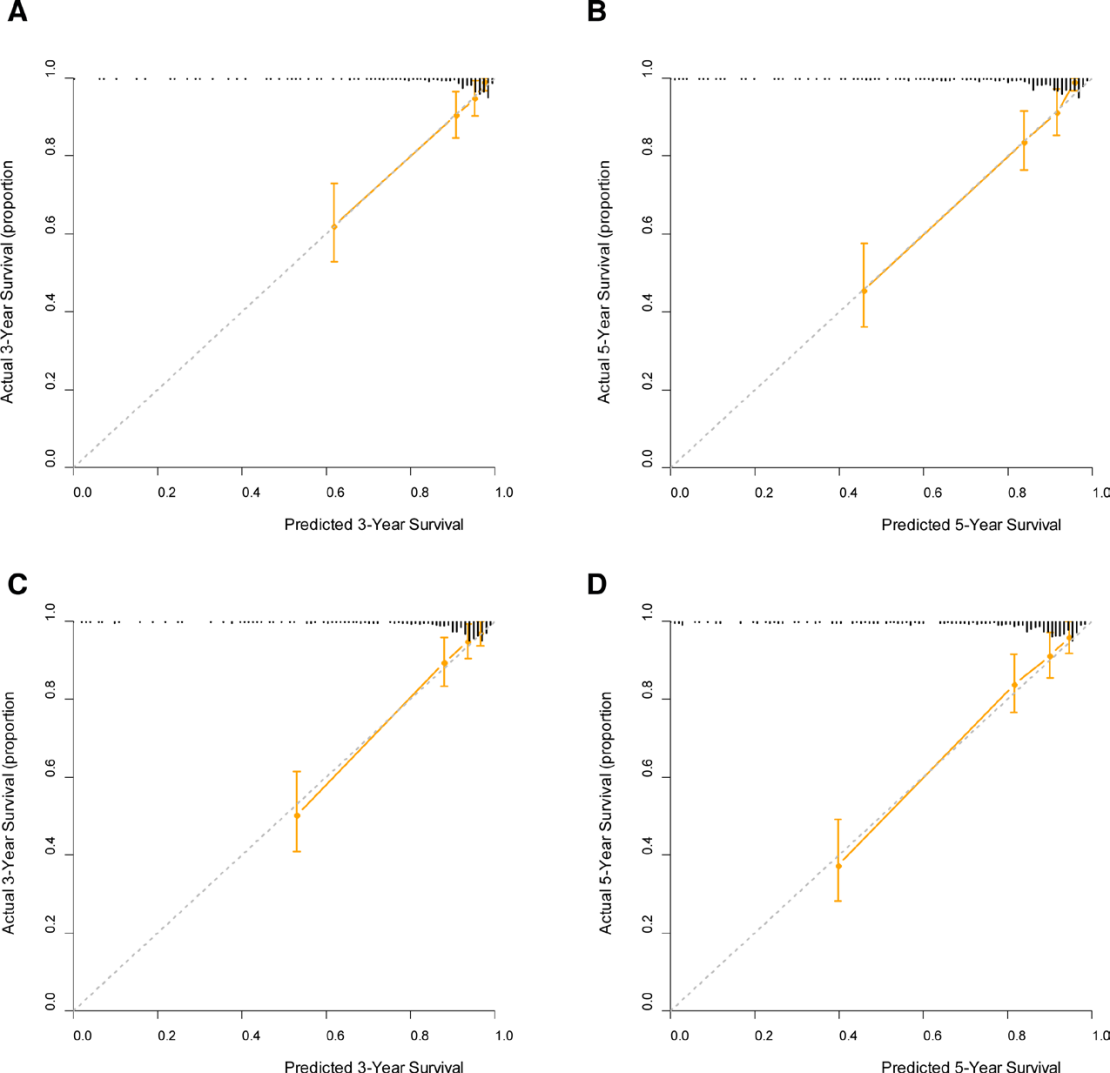
Calibration curve analysis of full model. (A) 3-year overall survival (OS) prediction, (B) 5-year OS prediction, (C) 3-year disease-free survival (DFS) prediction, and (D) 5-year DFS prediction. Full model = the model consisting of age, TNM stage, PNI, serum albumin level, and MLR. MLR = monocyte-to-lymphocyte ratio, PNI = perineural invasion, TNM = tumor–node–metastasis.

**Figure 3.**
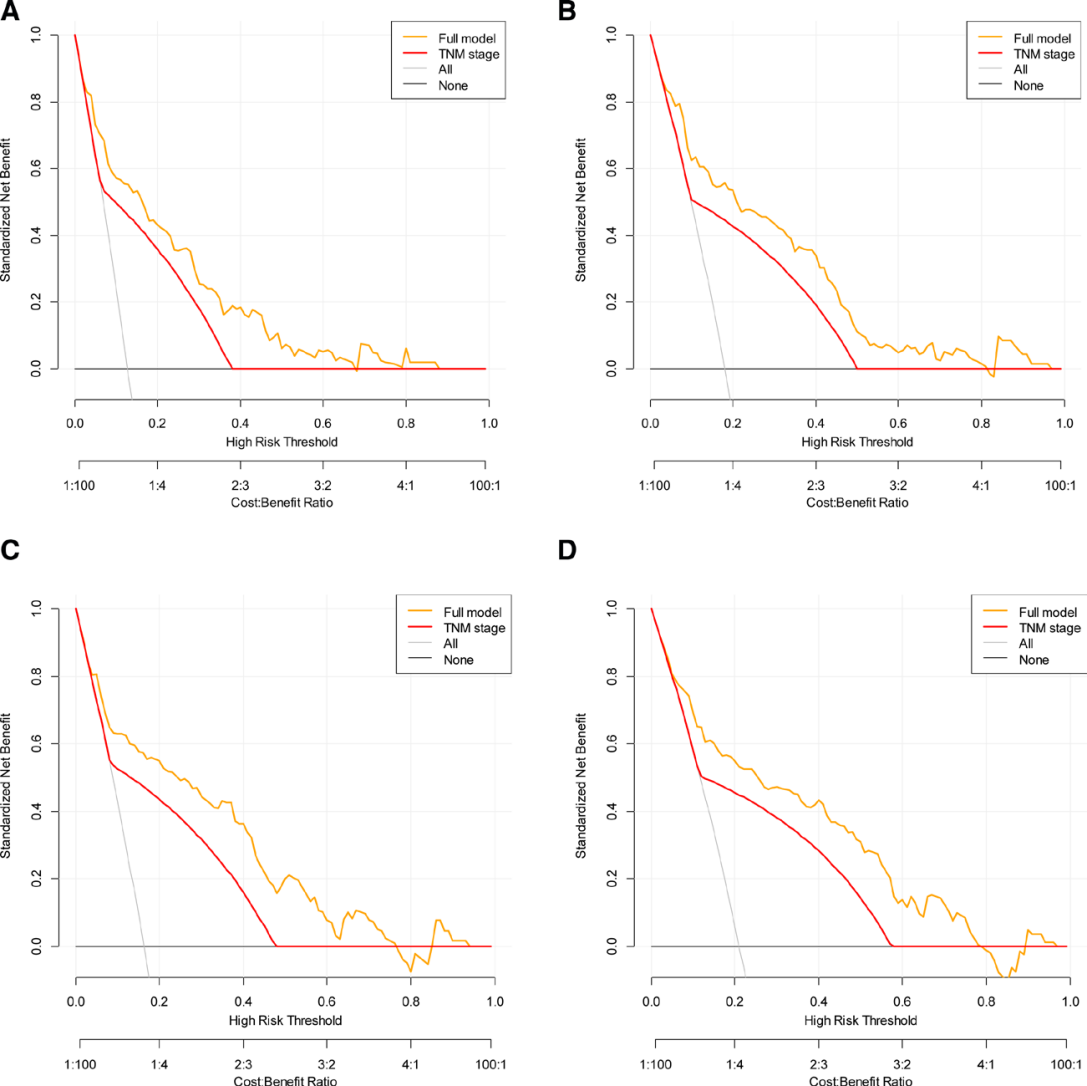
Decision curve analysis of the full model against tumor stage. (A) 3-year overall survival (OS) prediction, (B) 5-year OS prediction, (C) 3-year disease-free survival (DFS) prediction, and (D) 5-year DFS prediction. Full model = model consisting of age, TNM stage, PNI, serum albumin level, and MLR. MLR = monocyte-to-lymphocyte ratio, PNI = perineural invasion, TNM = tumor–node–metastasis.

Five covariates (i.e., age, TNM stage, PNI, ALB, and MLR) constituted the full model. Nomograms for predicting OS were established using the full model (Fig. 1). The calibration curves for the full model illustrated that the predicted survival closely matched the actual survival (Fig. 2). DCA for the models showed that the net benefits of the full model were higher than those of TNM across a range of nearly all thresholds (Fig. 3), supporting the better clinical value of the full model than that of TNM stage.

**Figure 4.**
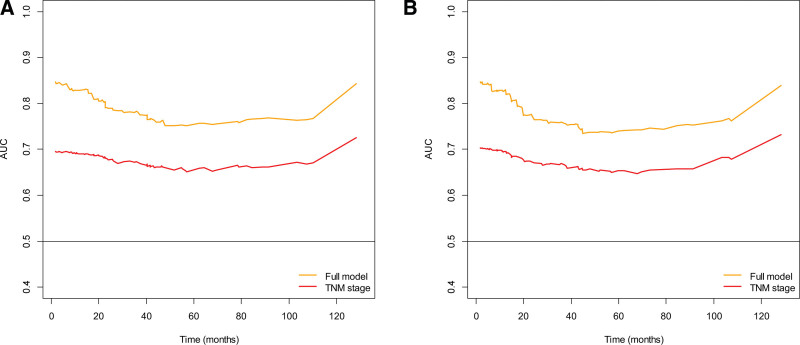
Time-varying prognostic accuracy of models over time using the incident/dynamic approach. (A) Overall survival, (B) disease-free survival. Full model = model consisting of age, TNM stage, perineural invasion, serum albumin level, and MLR. AUC = area under receiver operating characteristic, MLR = monocyte-to-lymphocyte ratio, TNM = tumor–node–metastasis.

The AUC (t) values of the full model for survival were larger than those of TNM stage at 36 and 60 months after surgical resection (Table 4). Additionally, the AUC (t) of the full model was found to be larger than that of TNM stage over a 10-year period after surgical resection (Fig. 4).

**Table 4:** AUC curve and Harrell concordance index of the full model against tumor stage.

Discrimination	Models		*P* value*
	Full model	TNM stage	
OS			
AUC (36 mo)	0.779	0.670	
AUC (60 mo)	0.754	0.656	
iAUC	0.792	0.677	
C-index	0.823	0.689	<.001
DFS			
AUC (36 mo)	0.763	0.664	
AUC (60 mo)	0.738	0.651	
iAUC	0.790	0.681	
C-index	0.813	0.695	<.001

AUC = area under receiver operating characteristic, C-index = Harrell concordance index, DFS = disease-free survival, Full model = the model consisting of age, TNM stage, perineural invasion, serum albumin level, and MLR, iAUC = integrated AUC, MLR = monocyte-to-lymphocyte ratio, OS = overall survival, TNM = tumor–node–metastasis.

* Between the full model and TNM stage.

The iAUCs of the models for OS were 0.792 for the full model and 0.677 for TNM stage. The iAUCs of the DFS models were 0.790 and 0.681 for the full model and the TNM stage, respectively (Table 4). The C-indices of the models for OS were 0.823 for the full model and 0.689 for TNM stage (*P* < .001). Additionally, the C-indices of the models for DFS were 0.813 and 0.695 for the full model and TNM stage, respectively (*P* < .001) (Table 4).

## 4. Discussion

This study showed that age, TNM stage, PNI, ALB, and MLR were significant determinants of both OS and DFS and constituted the full model. The predictive power of the full model was internally validated, and the full model was suggested to outperform TNM stage in predicting both OS and DFS.

MLR is considered an important prognostic determinant in diverse malignancies including hepatoma,^[15]^ gallbladder cancer,^[16]^ esophagogastric junction cancer,^[17]^ gastrointestinal stromal tumors,^[18]^ and colorectal cancer.^[19–21]^

With regard to GC, MLR has been reported to be a prognostic factor for survival in advanced-stage tumors undergoing chemotherapy.^[22–26]^ Similarly, MLR is a significant determinant of survival in patients with stage I to III GC.^[28–31]^ However, while MLR was a determinant of OS, DFS, or disease-specific survival in univariate analysis, the same was not true in multivariate analysis.^[28–31]^ Additionally, the results of previous studies are far from satisfactory enough to reach conclusions because the cutoff values of MLR are diverse, from 0.19 to 0.23, making it difficult to compare studies.^[27]^ Moreover, although dichotomizing a continuous variable may be useful for simplifying the analysis or transforming a skewed distribution into a more symmetrical distribution, using the optimal cutoff point from the receiver operating characteristic (ROC) curve analysis can result in overfitting if not carefully used.^[38,39]^

Therefore, in the present study, we treated MLR as a continuous variable and evaluated its clinical significance in patients with stage I to III GC. Treating variables as continuous allows for more flexible modeling of the relationships between variables and can help avoid the loss of information that can occur with dichotomization.^[38,39]^ In this study, MLR was proven to be a determinant of OS and DFS outcomes in multivariate analysis. In addition, the VIFs of MLR were 1.18 for OS and 1.20 for DFS, implying no significant collinearity. Therefore, the results of this study highlight the clinical importance of MLR as an independent predictor of survival in patients with stage I to III GC.

In the current study, in addition to MLR, age, TNM stage, PNI, and ALB were significant prognostic factors for OS and DFS in the multivariate Cox regression analysis.

These variables have been previously reported as prognostic factors in GC.^[40,41]^ Older patients may have reduced physiological reserves, comorbidities, and decreased response to treatment, leading to a higher risk of relapse or death. TNM staging is widely used as a standard for predicting the risk of future relapse or death.^[3]^ Low ALB is associated with malnutrition and systemic inflammation, leading to a poorer prognosis in GC.^[4,5]^

In this study, 5 covariates (i.e., age, TNM stage, PNI, ALB, and MLR) constituted the full model. When establishing nomograms to predict OS and DFS rates using the full model, age, ALB, and MLR were the main components of the overall scores, indicating their specific value as predictors of survival.

A calibration curve is a graphical tool used to evaluate the relationship between actual and predicted values. Ideally, a perfect calibration curve should demonstrate a linear relationship between 2 variables. In this study, we found that the slope of the calibration curve was close to the ideal value, indicating that the full model was well calibrated and accurately predicted the actual values. Moreover, the narrow 95% confidence interval suggests that the measurement system was highly precise and reliable. Overall, these findings further support the validity and robustness of the full model for predicting the survival outcomes of patients with GC.

DCA is a plot that shows the net benefit of a predictive model for a range of threshold probabilities.^[3,42]^ In this study, DCA with bootstrap analysis was performed to evaluate the clinical usefulness of the models. Our analysis showed that the full model had a higher net benefit for survival than TNM stage across almost all threshold probabilities. This suggests that the full model is clinically useful for predicting survival outcomes in patients with GC.

ROC curves show the relationship between sensitivity and 1-specificity for all possible thresholds of a continuous variable.^[2]^ However, time-dependent ROC curves may be more appropriate as disease outcomes are often time-dependent.^[42]^ In this study, we assessed the predictive performance of the full model and TNM stage for survival outcomes at 36 and 60 months after surgery using AUC (t). Our results showed that the AUC (t) of the full model was larger than that of TNM stage at the specified time points, indicating the superior predictive ability of the full model. Similarly, over a 10-year period after surgical resection, the AUC (t) of the full model was higher than that of the TNM stage for both OS and DFS, suggesting the superior predictive accuracy of the full model for long-term outcomes.

In this study, iAUC was used to measure the overall accuracy of a model in predicting the occurrence of a target event. The iAUCs of the full model for OS and DFS were 0.792 and 0.790, respectively, indicating excellent discrimination ability. Additionally, the full model demonstrated higher iAUCs for OS and DFS than TNM stage.

The C-index evaluates a model’s capability to predict the sequence of target events. In this study, the full model had a C-indices of 0.823 for OS and 0.813 for DFS, demonstrating excellent discrimination. Furthermore, the C-indices for OS and DFS were significantly higher in the full model than in TNM stage (*P* < .001, both). Therefore, the findings suggest that the full model is a superior predictor of survival compared to TNM stage.

The strengths of this study are as follows. First, while treating variables as continuous variables to avoid possible bias, we found that MLR was a significant determinant of OS and DFS in the multivariate analysis. Second, in addition to MLR, age, TNM stage, PNI, and ALB were important determinants of both OS and DFS, and constituted the full model. The full model was internally validated using bootstrapping. Additionally, the full model was suggested to outperform TNM stage in predicting both OS and DFS. The findings of this study have important clinical implications, as they could lead to more accurate risk stratification and individualized treatment planning for patients with GC. This information could be particularly useful for surgeons in identifying patients who are at a higher risk of poor survival outcomes, and could inform treatment decisions and postoperative follow-up plans. Further research should aim to externally validate the full model in independent patient cohorts to confirm its clinical utility and potential for widespread implementation.

The present study had several limitations that should be considered. First, because the study was conducted retrospectively, the possibility of missing data and selection bias could not be excluded, potentially impacting the generalizability of the results. Second, despite efforts to control for bias and random errors during study design and implementation, unmeasured or unknown confounders may have influenced the findings. Third, the study was limited to a single center, which may limit the generalizability of the results to other populations. Fourth, although the full model showed promising performance in predicting patient outcomes, further external validation is necessary before its widespread clinical implementation. Finally, while the sample size was adequate for the analyses performed, a larger sample size could potentially improve the precision of the estimates and increase the generalizability of the findings.

## 5. Conclusion

Age, TNM stage, PNI, ALB, and MLR were significant determinants of both OS and DFS, and constituted the full model. The full model was suggested to outperform TNM stage in predicting both OS and DFS. Therefore, our findings may help surgeons better differentiate patients with poor survival before gastrectomy. However, because MLR is a poorly characterized test for GC, these findings warrant further confirmation in large prospective clinical trials.

## Author contributions

**Conceptualization:** Soomin An, Wankyu Eo, Sookyung Lee, Yeong-Ju Lee.

**Data curation:** Soomin An, Wankyu Eo, Sookyung Lee, Yeong-Ju Lee.

**Formal analysis:** Soomin An, Wankyu Eo, Sookyung Lee.

**Funding acquisition:** Soomin An.

**Investigation:** Soomin An, Wankyu Eo, Sookyung Lee, Yeong-Ju Lee.

**Methodology:** Soomin An, Wankyu Eo, Sookyung Lee, Yeong-Ju Lee.

**Project administration:** Soomin An, Wankyu Eo, Sookyung Lee.

**Resources:** Soomin An, Wankyu Eo, Sookyung Lee.

**Software:** Soomin An, Wankyu Eo, Sookyung Lee.

**Supervision:** Wankyu Eo.

**Validation:** Soomin An, Wankyu Eo, Sookyung Lee.

**Visualization:** Soomin An, Wankyu Eo, Sookyung Lee.

**Writing – original draft:** Soomin An, Wankyu Eo.

**Writing – review & editing:** Soomin An, Wankyu Eo, Sookyung Lee, Yeong-Ju Lee.

## References

[1] Dittmar Y Schule S Koch A. Predictive factors for survival and recurrence rate in patients with node-negative gastric cancer-a European single-center experience. Langenbeck’s ArchSurg. 2015;400:27–35. 10.1007/s00423-014-1226-2 25048355

[2] Bansal A Heagerty PJ. A Tutorial on evaluating the time-varying discrimination accuracy of survival models used in dynamic decision making. Med Decis Making. 2018;38:904–16. 30319014 10.1177/0272989X18801312 PMC6584037

[3] Balachandran VP Gonen M Smith JJ. Nomograms in oncology: more than meets the eye. Lancet Oncol. 2015;16:e173–80. 25846097 10.1016/S1470-2045(14)71116-7 PMC4465353

[4] Akula B Doctor N. A prospective review of preoperative nutritional status and its influence on the outcome of abdominal surgery. Cureus. 2021;13:e19948. 34868791 10.7759/cureus.19948 PMC8627379

[5] An S Eo W Lee S. Comparison of the clinical value of the geriatric nutritional risk index and prognostic nutritional index as determinants of survival outcome in patients with gastric cancer. J Cancer. 2022;13:3348–57. 36186904 10.7150/jca.77397 PMC9516007

[6] Kiss M Caro AA Raes G. Systemic reprogramming of monocytes in cancer. Front Oncol. 2020;10:1399. 33042791 10.3389/fonc.2020.01399 PMC7528630

[7] Huang B Lei Z Zhao J. CCL2/CCR2 pathway mediates recruitment of myeloid suppressor cells to cancers. Cancer Lett. 2007;252:86–92. 17257744 10.1016/j.canlet.2006.12.012

[8] Arneth B. Tumor microenvironment. Medicina (Kaunas). 2019;56:15. 31906017 10.3390/medicina56010015 PMC7023392

[9] Eo WK Jeong DW Chang HJ. Absolute monocyte and lymphocyte count prognostic score for patients with gastric cancer. World J Gastroenterol. 2015;21:2668–76. 25759535 10.3748/wjg.v21.i9.2668 PMC4351217

[10] Okuno K Tokunaga M Yamashita Y. Preoperative lymphocyte-to-monocyte ratio is the most predictive inflammatory response marker of survival in gastric cancer. Langenbeck’s Arch Surg. 2021;406:2287–94. 34165594 10.1007/s00423-021-02230-9

[11] Kocak MZ Aktas G Duman TT. Monocyte lymphocyte ratio as a predictor of diabetic kidney injury in type 2 diabetes mellitus; The MADKID study. J Diabetes Metab Disord. 2020;19:997–1002. 33553019 10.1007/s40200-020-00595-0 PMC7843868

[12] Balci SB Aktas G. A Comprehensive review of the role of hemogram-derived inflammatory markers in gastrointestinal conditions. Iran J Colorectal Res. 2022;10:75–86.

[13] Tel BMA Bilgin S Kurtkulagi O. Frailty in diabetic subjects during COVID-19 and its association with HbA1c, mean platelet volume and monocyte//lymphocyte ratio. Clin Diabetol. 2022;11:119–26.

[14] Aktas G. Hematological predictors of novel Coronavirus infection. Rev Assoc Med Bras (1992). 2021;67(Suppl 1):1–2. 34259763 10.1590/1806-9282.67.Suppl1.20200678

[15] Wu Y Tu C Shao C. Inflammatory indexes in preoperative blood routine to predict early recurrence of hepatocellular carcinoma after curative hepatectomy. BMC Surg. 2021;21:178. 33794850 10.1186/s12893-021-01180-9 PMC8017621

[16] Choi YH Lee JW Lee SH. A high monocyte-to-lymphocyte ratio predicts poor prognosis in patients with advanced gallbladder cancer receiving chemotherapy. Cancer Epidemiol Biomarkers Prev. 2019;28:1045–51. 30842131 10.1158/1055-9965.EPI-18-1066

[17] Zhou WJ Wu J Li XD. [Effect of preoperative monocyte-lymphocyte ratio on prognosis of patients with resettable esophagogastric junction cancer]. Zhonghua Zhong Liu Za Zhi. 2017;39:178–83. 28316215 10.3760/cma.j.issn.0253-3766.2017.03.004

[18] Cananzi FCM Minerva EM Samà L. Preoperative monocyte-to-lymphocyte ratio predicts recurrence in gastrointestinal stromal tumors. J Surg Oncol. 2019;119:12–20. 30426498 10.1002/jso.25290

[19] Basile D Garattini SK Corvaja C. The MIMIC study: prognostic role and cutoff definition of monocyte-to-lymphocyte ratio and lactate dehydrogenase levels in metastatic colorectal cancer. Oncologist. 2020;25:661–8. 32202020 10.1634/theoncologist.2019-0780 PMC7418342

[20] Jakubowska K Koda M Grudzińska M. Monocyte-to-lymphocyte ratio as a prognostic factor in peripheral whole blood samples of colorectal cancer patients. World J Gastroenterol. 2020;26:4639–55. 32884222 10.3748/wjg.v26.i31.4639 PMC7445871

[21] Catal O Ozer B Sit M. The role of monocyte to lymphocyte ratio in predicting metastasis in rectal cancer. Ann Med Res. 2020;28:527–31.

[22] Fan X Wang D Zhang W. Inflammatory markers predict survival in patients with advanced gastric and colorectal cancers receiving Anti-PD-1 therapy. Front Cell Dev Biol. 2021;9:638312. 33791296 10.3389/fcell.2021.638312 PMC8005614

[23] Chen L Hao Y Zhu L. Monocyte to lymphocyte ratio predicts survival in patients with advanced gastric cancer undergoing neoadjuvant chemotherapy. OncoTargets Ther. 2017;10:4007–16. 10.2147/OTT.S140118 PMC5558583 28860808

[24] Zhou D Wu Y Zhu Y. The prognostic value of neutrophil-to-lymphocyte ratio and monocyte-to-lymphocyte ratio in metastatic gastric cancer treated with systemic chemotherapy. J Cancer. 2020;11:4205–12. 32368303 10.7150/jca.39575 PMC7196266

[25] Song S Li C Li S. Derived neutrophil to lymphocyte ratio and monocyte to lymphocyte ratio May be better biomarkers for predicting overall survival of patients with advanced gastric cancer. OncoTargets Ther. 2017;10:3145–54. 10.2147/OTT.S138039 PMC5495088 28706446

[26] Chen L Yan Y Zhu L. Systemic immune-inflammation index as a useful prognostic indicator predicts survival in patients with advanced gastric cancer treated with neoadjuvant chemotherapy. Cancer Manag Res. 2017;9:849–67. 29276407 10.2147/CMAR.S151026 PMC5733921

[27] Schiefer S Wirsik NM Kalkum E. Systematic review of prognostic role of blood cell ratios in patients with gastric cancer undergoing surgery. Diagnostics (Basel). 2022;12:593. 35328146 10.3390/diagnostics12030593 PMC8947199

[28] Li S Lan X Gao H. Systemic Inflammation Response Index (SIRI), cancer stem cells and survival of localized gastric adenocarcinoma after curative resection. J Cancer Res Clin Oncol. 2017;143:2455–68. 28828692 10.1007/s00432-017-2506-3 PMC11819166

[29] Liu Z Ge H Miao Z. Dynamic changes in the systemic inflammation response index predict the outcome of resettable gastric cancer patients. Front Oncol. 2021;11:577043. 33718137 10.3389/fonc.2021.577043 PMC7947713

[30] Shi H Jiang Y Cao H. Nomogram based on systemic immune-inflammation index to predict overall survival in gastric cancer patients. Dis Markers. 2018;2018:1787424. 30627220 10.1155/2018/1787424 PMC6305021

[31] Feng F Sun L Zheng G. Low lymphocyte-to-white blood cell ratio and high monocyte-to-white blood cell ratio predict poor prognosis in gastric cancer. Oncotarget. 2017;8:5281–91. 28029656 10.18632/oncotarget.14136 PMC5354908

[32] Mranda GM Xue Y Zhou XG. Revisiting the 8th AJCC system for gastric cancer: a review on validations, nomograms, lymph nodes impact, and proposed modifications. Ann Med Surg (Lond). 2022;75:103411. 35386808 10.1016/j.amsu.2022.103411 PMC8977912

[33] Harrison P Goodall AH. Studies on Mean Platelet Volume (MPV) - new editorial policy. Platelets. 2016;27:605–6. 27612027 10.1080/09537104.2016.1225467

[34] Noris P Melazzini F Balduini CL. New roles for mean platelet volume measurement in the clinical practice? Platelets. 2016;27:607–12. 27686008 10.1080/09537104.2016.1224828

[35] Harrell FE Jr. Lee KL Mark DB. Multivariable prognostic models: issues in developing models, evaluating assumptions and adequacy, and measuring and reducing errors. Stat Med. 1996;15:361–87. 8668867 10.1002/(SICI)1097-0258(19960229)15:4<361::AID-SIM168>3.0.CO;2-4

[36] Pencina MJ D’Agostino RB. Overall C as a measure of discrimination in survival analysis: model specific population value and confidence interval estimation. Stat Med. 2004;23:2109–23. 15211606 10.1002/sim.1802

[37] Kang L Chen W Petrick NA. Comparing two correlated C indices with right-censored survival outcome: a one-shot nonparametric approach. Stat Med. 2015;34:685–703. 25399736 10.1002/sim.6370 PMC4314453

[38] Altman DG Royston P. The cost of dichotomizing continuous variables. BMJ. 2006;332:1080. 16675816 10.1136/bmj.332.7549.1080 PMC1458573

[39] Royston P Altman DG Sauerbrei W. Dichotomizing continuous predictors in multiple regression: a bad idea. Stat Med. 2006;25:127–41. 16217841 10.1002/sim.2331

[40] Eo W Kwon J An S. Clinical significance of paraspinal muscle parameters as a prognostic factor for survival in gastric cancer patients who underwent curative surgical resection. J Cancer. 2020;11:5792–801. 32913472 10.7150/jca.46637 PMC7477437

[41] Eo WK Chang HJ Suh J. The prognostic nutritional index predicts survival and identifies aggressiveness of gastric cancer. Nutr Cancer. 2015;67:1260–7. 26583916 10.1080/01635581.2015.1082112

[42] Son SH Kang SM Jeong SY. Prognostic value of volumetric parameters measured by pretreatment 18F FDG PET/CT in patients with cutaneous malignant melanoma. Clin Nucl Med. 2016;41:e266–73. 27055144 10.1097/RLU.0000000000001205

